# Executing Learning Activities and Autonomy-Supportive Instructions Enhance Autonomous Motivation

**DOI:** 10.3389/fpsyg.2020.02109

**Published:** 2020-08-21

**Authors:** Paul Hinnersmann, Katrin Hoier, Stephan Dutke

**Affiliations:** Institute for Psychology in Education, University of Münster, Münster, Germany

**Keywords:** motivational changes, autonomous motivation, autonomy-support, cognitive dissonance, flow

## Abstract

This study investigated situational changes in learners’ degree of autonomous regulation during other-initiated learning activities and examined the influence of the instructional style on such changes. To this end, relative autonomous motivation of 172 fifth to seventh grade students was measured before, during and after execution of a musical learning activity. It was experimentally manipulated whether students were instructed in an autonomy-supportive or a controlling style. As expected based on self-determination theory and the action-based model of cognitive dissonance, relative autonomous motivation increased in the course of the execution of the learning activity. Unexpectedly, this increase was only statistically significant when students were instructed in a controlling style. At all times though, students instructed in an autonomy-supportive style were more autonomously motivated than students instructed in a controlling style. Furthermore, results showed a positive effect of an autonomy-supportive instructional style on students’ functional state and their interest in continuing with the learning activity. The pattern of changes in relative autonomous motivation might indicate that in controlling conditions a reduction of dissonance is of functional importance, which is why relative autonomous motivation increased under controlling conditions but not under autonomy-supportive conditions. In an applied perspective, the study demonstrates that executing an activity might be beneficial for fostering autonomous motivation and it corroborates findings that indicate positive effects of an autonomy-supportive instructional style on students’ motivation and functional state.

## Introduction

In this study, short-term changes in the quality of motivation within learning activities are the matter of interest. Based on self-determination theory (Deci and Ryan, 1985, 2000; Ryan and Deci, 2017) and the action-based model of cognitive dissonance (Harmon-Jones et al., 2015) it is assumed that in other-initiated learning activities relative autonomous motivation increases during the execution of learning activities when the situation is autonomy-supportive. Broadening the knowledge about short-term motivational changes is important for understanding motivational dynamics in class. This understanding might help instructors to be able to diagnose the motivational state of their students more accurately and foster beneficial types of motivation in students more systematically.

Motivation changes in the course of time (Atkinson and Birch, 1970). This dynamic nature of motivation is necessary for people to adapt their behavior according to external and internal situational demands (Schultheiss and Wirth, 2010). At the same time, stability in people’s behaviors is of necessity, too. If each arising action tendency was followed immediately in situations of motivational conflict, a single activity could not be carried out efficiently since there would be permanent changes in the flow of action, presumably without achieving the goal (Atkinson and Birch, 1970). Therefore, volition or “realization motivation” (Kuhl, 1983) is needed to ensure that a behavior is continued even when obstacles occur. Kuhl (1987) differentiated several volitional strategies. One of them is to increase motivation for implementing the behavior one has decided to execute. Here, the individual enhances the value of the activity for him- or herself. Thereby, the motivation changes in the course of the execution of the activity while the activity stays the same. This kind of motivational change in the course of the execution of a singular activity is the focus of the present study. Specifically, changes in relative autonomous motivation are investigated.

Relative autonomous motivation is a construct that stems from self-determination theory (Ryan and Connell, 1989; Roth et al., 2007), in which several types of motivation are differentiated (Deci and Ryan, 2000). There is the distinction of intrinsic and four different kinds of extrinsic motivation. Intrinsically motivated behavior is understood as acting because of the satisfaction inherent in the execution of an action itself (Ryan and Deci, 2000a). It is seen as a tendency of engaging in challenging and interesting activities, whereby competences and knowledge are developed (Vansteenkiste et al., 2010). In contrast, extrinsically motivated behavior is thought of as being executed because of the consequences resulting from executing that behavior. Of course, intrinsically motivated behaviors result in consequences, too, such as new competences and knowledge. But while extrinsically motivated behaviors are executed in order to obtain the desired consequences, intrinsically motivated behaviors are executed for their own sake. So, while intrinsic motivation can be thought of as “autotelic” in nature, extrinsic motivation can be thought of as “instrumental” in nature (Deci and Ryan, 1993, p. 225).

Based on the study of the attributes of intrinsic and the different kinds of extrinsic motivation, self-determination theory arrived at differentiating between autonomous (or self-determined) forms of motivation and controlled (or other-determined, heteronomous) forms of motivation which is the main focus of differentiation in self-determination theory nowadays (Deci and Ryan, 2008). According to self-determination theory, the different types of motivation can be located on a continuum of relative autonomy (Ryan and Connell, 1989). Thus, the different types of motivation – whether it is intrinsic motivation or a type of extrinsic motivation – can be differentiated based on the degree of experienced autonomy characteristic of each particular type of motivation. When being autonomously motivated, a behavior is executed willingly and volitionally. This is because a person enjoys the activity or identifies with and approves and appreciates the behavior in question when being autonomously motivated (Deci and Ryan, 2008). In contrast, behaviors based on controlled motivation are characterized by resting upon external or internal pressures to act in certain prescribed ways (Deci and Ryan, 2008).

The term relative autonomous motivation reflects that a behavior can be motivated by several different reasons and thus by different autonomous and controlled types of motivation at the same time. Accordingly, relative autonomous motivation is the sum of the different reasons a behavior is motivated by. Therefore, it expresses to what degree a behavior is autonomously or heteronomously motivated on a continuum ranging from self-determination to other-determination. Although conceptually identical, relative autonomous motivation has also often been operationalized under the terms of relative autonomy index (e.g., Grolnick and Ryan, 1987) or self-concordance index (e.g., Koestner et al., 2002).

To date, research on changes in the quality of motivation has mainly focused on rather long periods of time (weeks, months, and years) in the educational field (e.g., Otis et al., 2005; Tsai et al., 2008; Cheon et al., 2012; Kyndt et al., 2015). A robust finding from cross-sectional and longitudinal studies is that autonomous motivation of students – mostly investigated with regard to intrinsic motivation – declines during their school career. This has been shown for elementary schools (e.g., Spinath and Spinath, 2005; Spinath and Steinmayr, 2008), in the transition from elementary school to high school (e.g., Harter, 1981), though here the effect might not apply to all school subjects (Gottfried et al., 2001), and within the high school years (e.g., Otis et al., 2005; Leroy and Bressoux, 2016). Mixed results have been found with regard to controlled forms of motivation. Whereas Leroy and Bressoux (2016) found that students’ controlled motivation also decreased in the first year of junior high school and Otis et al. (2005) reported the same results for students from 8th to 9th grade, Nishimura and Sakurai (2017) found an increase in controlled motivation among students from 7th to 9th grade. In line with the latter study, Buff (2001) found that 11th graders were more likely to be other-determined motivated compared to elementary school students and that elementary school students were more likely to be self-determined motivated compared to 11th graders. Similarly, in a person-centered approach Hayenga and Corpus (2010) found changes in middle-school students who developed from being rather autonomously motivated to being rather controlled motivated. However, Corpus and Wormington (2014), also using a person-centered approach, found that a substantial amount of primarily intrinsically motivated elementary school students stayed intrinsically motivated in the course of about half a year. Changes in amotivation – meaning not having any intention to act – have been less often investigated. Leroy and Bressoux (2016) reported an increase in students’ amotivation in high-school. Summarized, these results, with some exceptions (Gottfried et al., 2001; Corpus and Wormington, 2014), indicate a trend toward a decrease in relative autonomous motivation during school education.

Beyond such long-term changes in motivation, experimental studies demonstrated that the quality of motivation can also be influenced in short periods of time measured in minutes (e.g., Deci et al., 1994; Jang, 2008). However, it is widely unknown whether and how the quality of motivation changes during the execution of a learning activity. Moreover, motivational changes taking place in short periods of time probably do not follow regularities similar to those observed in the studies on long-term changes. Therefore, in the present study, we investigate motivational changes in short periods of time during other-initiated learning activities.

Other-initiated learning activities are understood as situations in which an instructor prompts students to execute a specific learning activity, a situation that takes place frequently in educational settings, for example, a music teacher asking students to analyze a certain piece of classical music. In such situations, learners might experience cognitive dissonance. A pivotal assumption in self-determination theory is that people strive after satisfying the three basic psychological needs for autonomy, competence, and relatedness in order to foster their well-being and personal development (Deci and Ryan, 2000; Ryan and Deci, 2000a). Most relevant in the context of this study is the need for autonomy, which can be described as “the need of individuals to experience self-endorsement and ownership of their actions” (Ryan and Deci, 2017, p. 86). In other-initiated learning activities the reason of acting because of the external request is salient. Such a reason is indicative of controlled motivation and thus potentially a situation in which the need for autonomy is rather frustrated than satisfied. Hence, in other-initiated learning activities, potentially there is a dissonance between perceiving oneself as acting due to controlled motivation and the need for autonomy. According to the action-based model of cognitive dissonance (Harmon-Jones et al., 2015), people strive after resolving such inconsistencies in order to be able to execute the behavior at hand efficiently and without conflict. To do this, “individuals change their attitudes to be consistent with their behavior” (Harmon-Jones and Harmon-Jones, 2002, p. 712). In the situation of other-initiated learning activities, this can be achieved for example by finding value, personal benefit, interestingness and/or enjoyability in the action or by weighing the value, personal benefit, interestingness and/or enjoyability one sees in the action more strongly. For example, students who analyze a piece of music might find out that this activity helps them to understand the music better, makes them find meanings in it that are of some importance to their personal lives or that it is even fun to think about music in this analytical way. Thus, dissonance in other-initiated learning activities would be reduced here by increasing the relative autonomous motivation for that action. Therefore, since individuals are motivated to reduce dissonance (Festinger, 1957), relative autonomous motivation should increase in the course of the execution of an other-initiated learning activity.

The same would be expected based on self-determination theory which hypothesizes that individuals tend toward adopting values and regulations of others, thereby integrating themselves into the social community and developing a congruent self (Ryan, 1993; Sheldon and Kasser, 1995; Deci and Ryan, 2000; Baumann, 2009; Weinstein et al., 2013). Due to this internalization process, externally regulated behaviors can change into self-regulated behaviors. In other words, a behavior that is executed based on controlled motivation can change to be executed based on autonomous motivation. However, although claimed to be innate, the tendency to internalize the regulation of behavior depends on environmental factors and, therefore, can be altered in quality and magnitude by the social context (e.g., Deci et al., 1994; Reeve et al., 2002). The same applies for intrinsic motivation. Here, too, environmental factors potentially influence the magnitude of intrinsic motivation for actions (Deci et al., 1999). Therefore, for internalization to take place and intrinsic motivation to flourish, the basic psychological needs for autonomy, competence, and relatedness need to be satisfied by the social environment (Deci and Ryan, 1985; Deci et al., 1994; Ryan and Deci, 2000a).

In self-determination theory autonomy-supportive contexts are contrasted with controlling contexts. Autonomy-supportive contexts are thought of as contexts in which authority figures (e.g., instructors) rely on nurturing the inner motivational resources of their subordinates (e.g., learners) for fostering their motivation by addressing the psychological needs of the subordinates, by using informational language, by giving explanatory rationales, and by acknowledging and accepting negative affect of subordinates (Cheon et al., 2012). In contrast, controlling contexts are thought of as contexts in which authority figures rely on extrinsic sources of motivation (e.g., offering incentives or threatening with negative consequences), rely on controlling language, provide no explanatory rationales, and try to change negative affect of subordinates (Cheon et al., 2012). Autonomy-supportive contexts provide conditions in which intrinsic motivation is likely to flourish (e.g., Jang et al., 2009) and in which successful internalization is likely to take place (Deci et al., 1994). In contrast, controlling contexts are likely to undermine intrinsic motivation (Deci et al., 1999) and impede internalization (Deci et al., 1994). Because of these effects on intrinsic motivation and internalization we hypothesize that relative autonomous motivation will increase in the course of the execution of an action if initiated by another person in an autonomy-supportive style but not when initiated in a controlling style.

Although the present study focuses on motivational changes, we additionally investigated the relationship between relative autonomous motivation and (a) students’ functional state during the learning activity and (b) their interest in continuing with the learning activity. Functional state refers to the degree to which learners can make use of their capacities for optimal learning at a given moment. It is characterized inter alia by how absorbed the learner is in the learning activity at hand and by the extent of worrying thoughts being present. As predicted by self-determination theory and supported by empirical findings (e.g., Grolnick and Ryan, 1987; Lee et al., 2003; Vansteenkiste et al., 2004, 2005; Evans and Bonneville-Roussy, 2016; Valenzuela et al., 2018) positive correlations are expected between relative autonomous motivation and the students’ functional state during the learning activity and between relative autonomous motivation and students’ interest in continuing with the learning activity later on.

Also, we looked at the effects of autonomy-supportive and controlling instructional behavior on students’ relative autonomous motivation overall, students’ functional state measured by their experience of flow and degree of worrying, and the students’ interest in continuing with the learning activity after lesson. Based on previous findings, it is hypothesized that, when being instructed in an autonomy-supportive style compared to being instructed in a controlling style, students will on average (a) show higher relative autonomous motivation (e.g., Pelletier et al., 2001; Vansteenkiste et al., 2012; Evans and Bonneville-Roussy, 2016), (b) will function more optimally during the learning activity (e.g., Assor et al., 2002; Vansteenkiste et al., 2005; Reeve and Tseng, 2011), and (c) will be more interested in continuing with the learning activity (e.g., Vansteenkiste et al., 2004; Tsai et al., 2008; Bonneville-Roussy et al., 2013; Bonneville-Roussy et al., 2020).

Summarized, it is predicted:

H1: Relative autonomous motivation increases in the course of the execution of an other-initiated learning activity when the activity is instructed in an autonomy-supportive style and does not increase when it is instructed in a controlling style.
H2: Relative autonomous motivation is higher in autonomy-supportive situations compared to controlling situations.
H3: Relative autonomous motivation correlates positively with the functional state of learners in terms of lower worrying and higher flow tendencies.
H4: Students’ functional state is more positive in autonomy-supportive situations compared to controlling situations in terms of lower worrying and higher flow tendencies.
H5: Relative autonomous motivation and instructional style (autonomy-supportive vs. controlling) predict the interest in continuing with the learning activity.

To test these hypotheses, relative autonomous motivation of fifth- to seventh-graders for a particular learning activity was measured repeatedly during lesson. Students in their regular music lessons were instructed by an experimenter to learn and execute diverse rhythms in the group using their body (body percussion) or their voice (vocussion). Instructions were either given in an autonomy-supportive style or a controlling style. Before, during and after the execution of these rhythm exercises students’ motivation for executing the rhythm exercises was measured. Additionally, during the execution of the rhythm exercises students’ degree of worrying and of experiencing flow was measured and after the execution of the rhythm exercises students’ interest in continuing with this kind of learning activity later on was measured.

## Materials and Methods

### Sample

One hundred and seventy-four fifth- to seventh-graders participated in this study. The data of two students were excluded from analyses because they were late for class and missed the first assessment. Of the remaining *N* = 172 participants *n* = 51 (29.7%) were in fifth grade, *n* = 74 (43%) in sixth grade, and *n* = 47 (27.3%) in seventh grade. Of the students *n* = 95 were female (55.2%), *n* = 74 students were male (43%), and three students did not indicate their gender (1.7%). Age ranged between 10 and 14 years (*M* = 11.55, *SD* = 0.97). Eighty-one of the students (47.1%) indicated that they played a musical instrument and 95 students (55.2%) indicated that they were able to read music well or very well. Students either attended a grammar school (“Gymnasium,” *n* = 40; 23.3%) or a comprehensive school (“Gesamtschule,” *n* = 132; 76.7%). Grammar schools and comprehensive schools are schools of the secondary school system in Germany. Grammar schools prepare students for university education. Comprehensive schools can prepare students for university education as well but also prepare for vocational training. With regard to the distribution of students into the two experimental conditions (autonomy-supportive vs. controlling instructional style), there was no significant difference between students attending grammar school (47.5% vs. 52.5%) and students attending comprehensive school (38.6% vs. 61.4%), *X*^2^(1) = 1.00, *p* = 0.32. Music is taught in elementary school and all participating students attended the compulsory music lessons at their school at the time the study took place. Thus, it can be reasoned that all students in the study had had musical training to some degree.

The participating schools were informed about the procedures and goals of the study and approved it. Participants were informed about the procedure of the study beforehand and were thoroughly debriefed about the experimental manipulation after the study. In their ethical guidelines, the Federation of the German Psychologists’ Associations (2016) declared that researchers can abstain from obtaining informed consent from participants, if participation is reasonably not harmful to subjects and if research refers to common teaching situations in educational settings. Since this applies to this study written consent was not requested from participants and an ethical review and approval by an ethics committee was not required.

### Research Design

A 2 × 3 factorial design was employed, with the independent variables instructional style (autonomy-supportive vs. controlling) as a between-subject variable and time of assessment (before, during, and after execution of the rhythm-exercise) as a within-subject variable. Degree of relative autonomous motivation is the dependent variable with regard to Hypotheses 1 and 2. Hypothesis 3 is tested by means of a correlation analysis. Therefore, the included variables are not classified as dependent or independent variables. With regard to Hypothesis 4 instructional style is the independent variable and flow and worrying are the dependent variables. With regard to Hypothesis 5, interest in continuing with the rhythm exercises is regressed on the independent variables relative autonomous motivation and instructional style.

### Procedure

The study took place at the time of students’ regular music lessons in their regular classes. In the experimental situation, classes were not instructed by the students’ regular teachers, but all classes were taught by the second author of this paper. She was unknown to the students before the study. Sessions lasted about 60 min.

In the beginning of each class, the experimenter introduced herself and informed students about the procedure. Students answered a sample questionnaire to get acquainted with the format of the questions used in this study. After all questions of the students concerning the procedure and the questionnaires were answered, the experimental manipulation started. Students were either introduced to the learning content of the lesson in an autonomy-supportive instructional style or in a controlling style and were instructed in the same style throughout until the end of the learning unit. The content of the lessons was learning how to make rhythms in a group using one’s body (body percussion) and voice (vocussion). A teaching method called “live-arrangement” by Terhag and Winter (2011) was used in this study. Here, based on a (rhythmic) core pattern produced by the whole group of learners, individual students or subgroups of students successively learn new patterns that are variations of and that go along with the core pattern. The whole class first learned body percussion patterns and vocussion patterns and variations thereof that were provided by the teacher. Later on, they were also asked to create own body percussion and vocussion patterns. The musical exercises were identical in both experimental conditions. All rhythmic patterns were in four–four time.

In detail, the following exercises took place during the lesson. First, students walked through the classroom in different styles (e.g., slow, as silently as possible) and after about 5 min were supposed to find a joint walking pace as a group. The instructor then counted in four–four time in the students’ pace of walking. While walking through the room, students were then gradually instructed to stamp once with their feet on the first beat, to clap once on the second beat, to click their fingers on the third beat and to clap twice on the fourth beat. This activity lasted about 5 min. Then students sat down on chairs and the instructor and students alternated with performing rhythms. The instructor started with a rhythmic pattern like clapping once on the first and second beats, clapping twice on the third beat, and clapping once on the fourth beat and the students repeated that pattern in the pace of the teacher. Then the instructor varied by using another part of the body to clap on for some of the beats of the bar or by also clapping twice on the second beat etc. After about 4 min, students were invited (autonomy-supportive condition) or asked (controlling condition) to take over the part of the instructor and perform rhythmic patterns that the remaining students then had to reproduce. If no student took over the part of the instructor, the instructor kept on showing rhythms that the students then reproduced. After about 3 min, the students learned rhythmical vocussion core patterns and successively learned new variations that went along with the core patterns. This activity lasted about 9 min and was the last activity of the lesson.

The teaching method is particular in that skills on a musical instrument are not needed for participating, that students immediately perform music together while practicing rather than first practicing individually on their own before performing music together, and that the level of difficulty can easily be adjusted to an individual’s skill level. In ordinary music lessons, these features are also realized within different teaching methods and regarding different learning contents (e.g., singing songs together in class). Therefore, the lesson conducted in the framework of this study can be seen as a rather regular practical music lesson, fitting in with the curriculum of music education in comprehensive schools and grammar schools in Germany (e.g., Ministry for School and Further Education North Rhine-Westphalia, 2012, 2019).

Students answered questionnaires three times during the lessons – before (t1), during (t2), and after execution of the learning activity (t3). At all three times of assessment students’ relative autonomous motivation was measured. At t2 students additionally indicated their experience of flow and worrying during the learning activity. At t3 students additionally rated their interest in continuing with this kind of learning activity at a later time and gave some demographic information. In a final step after completion of all questionnaires, students were thoroughly debriefed about the aims of the study and the experimental manipulation.

### Experimental Manipulation

For establishing an autonomy-supportive and a controlling situation, the experimenter implemented different behaviors derived from Reeve (2009). In the autonomy-supportive condition, it was aimed at motivating students by building upon their inner motivational resources by means of addressing the psychological needs of autonomy, relatedness, and competence (Cheon et al., 2012). In the controlling condition, it was aimed at motivating students based on social pressure to act in certain prescribed ways. Since there is evidence that only applying different autonomy-supportive behaviors in a coordinated way is effective in initiating the process of internalization (Deci et al., 1994), several autonomy-supportive behaviors and controlling behaviors, respectively, were used.

#### Autonomy-Supportive Instructional Style

In introducing the learning activity at the beginning of the lesson, the experimenter provided the students with a rationale for why practicing rhythm exercises might be worthwhile for them. This was realized by (a) asking students about their ideas why these exercises might be useful for them and (b) naming benefits that might come along with exercising rhythms if students did not mention them themselves (e.g., learning to listen to each other, improving one’s coordination abilities). The experimenter also emphasized that the exercises might be fun. In the course of the learning activity the experimenter explained why students were asked to do certain activities in a certain way or what they might learn from the exercises (e.g., sitting upright on the chair because this is helpful when making music; explaining that they will learn making rhythms with different parts of their body). Thus, students were provided with rationales during the learning activity as well. During the execution of the rhythm exercises students were allowed and asked to bring in own ideas (e.g., walking in a certain way during a warm-up game or performing rhythms invented by themselves). Thereby, the experimenter emphasized the possibility to make choices and to take the initiative. Throughout the lesson, the experimenter avoided the use of controlling language (e.g., saying “must,” “have to,” “should” etc.) and regularly gave positive and informative feedback (e.g., “This worked very well because we listened to each other.”). If students were reluctant to perform single rhythm tasks, the students’ presumably negative feelings toward this activity were validated and a decision not to perform the task was accepted (e.g., “I understand if you do not have the heart for this. After all you do not know me that well yet.”).

#### Controlling Instructional Style

In this condition, no rationale for why practicing rhythm exercises might be worthwhile was given. Instead, the experimenter emphasized that the students had to make an effort and stay concentrated in order to avoid failure (e.g., “Today you have to create short rhythmical pieces. If you do not apply yourselves and follow the exercises precisely, it will not work. Therefore concentrate, please!”). Students were offered no choices as to bring in own ideas. Initiatives for own actions were inhibited. Instead, students were requested to follow the instructions of the experimenter. If students were reluctant to perform individual rhythm tasks, the experimenter demanded following her instructions (e.g., “Just try it! It is not that hard.”). Throughout the lessons, the experimenter attempted to use pressure-inducing, controlling language (e.g., saying “must,” “have to,” “should” etc.). Explicit feedback was not given in the controlling condition.

### Measures

#### Relative Autonomous Motivation

For measuring the degree of students’ relative autonomous motivation an approach introduced by Buff (2001) and Buff et al. (2010) was used in a slightly adapted and extended version. Students indicated their reasons on the open-ended question for why they were executing the rhythm exercises. Depending on when they were to answer this question (t1, t2, or t3), the question slightly differed (t1: “When you are executing the rhythm exercises in a moment’s time, why will you be doing this?”; t2: “Why are you executing the rhythm exercises right now?”; t3: “Why did you just execute the rhythm exercises?”). After answering these questions, the students were prompted to write down their current reasons in their own words. They were allowed to write down four reasons at most. Four lines were placed under the question and students were asked to use one line for each reason. This approach to measure motivation was chosen because we did not know which reasons students might have for executing the learning activity at the three different times of measurement. Therefore, we did not want to provide them with a questionnaire presenting predefined reasons. By using such an approach, reasons relevant to the students might have been missed.

Next, students were asked to mark the importance of each of their reasons for executing the rhythm exercises on a 7-point Likert scale (ranging from “not important at all” to “extremely important”). These scales were placed in each row on the right-hand side of the lines on which students wrote down their reasons. The importance ratings were included because not only might reasons change in the course of time but also (or only) their importance.

The reasons and importance ratings were used to calculate an index of relative autonomous motivation corresponding to the approach of Buff et al. (2010). First, the reasons were categorized independently by two trained raters. A category system was used that comprised 15 categories of which one is a residual category (see Appendix). Eleven categories were taken from the category system by Buff et al. (2010). Three categories were added: (a) “benefit for others,” (b) “testing one’s own skills,” and (c) “completing things one began with.” In unpublished pilot studies, these categories crystalized as important for categorizing reasons for the execution of a particular activity in contrast to reasons for engagement in a certain school subject in general, which was the focus of the study by Buff et al. (2010). Initial interrater reliability across all 15 categories was good with *K* = 0.80. In a second categorization process, all reasons the raters initially ascribed to different categories, were re-analyzed together with a third rater. If all raters agreed on one category for this reason, it was re-categorized. By means of this process, interrater reliability increased to *K* = 0.99. All reasons for which no consensus was reached, were excluded from the analyses (0.90%), as were answers that did not constitute reasons but rather descriptions of the executed activity (1.35%) and reasons that were ascribed to the residual category (3.17%).

Second, the categories were rated on a 6-point Likert scale by five experts of self-determination theory with regard to the extent that reasons falling into one category indicated autonomous versus controlled motivation. Thus, the mean of these ratings per category indicates the degree to which the reasons belonging to the particular category indicate autonomous motivation. This indicator is referred to as the *reference value*. The reference value varies between values of −2.5 and 2.5.

Third, the following formula was used to calculate the *index of relative autonomous motivation*:

$$\frac{\sum Referencevalue\times Importancevalue}{Numberofstatedreasons}$$

For each student, three indices of relative autonomous motivation were calculated, one for each of the three measurements t1 to t3. For each reason, the corresponding reference value – resulting from the category this reason was ascribed to – was multiplied by the corresponding importance value the student had ascribed to this reason. The products for all reasons the student mentioned in the respective point of measurement were summed up and divided by the number of reasons given at the particular time of measurement (t1, t2, or t3). Since the maximum of the importance value is 7 and the reference value’s minimum is −2.5 and it’s maximum 2.5 the theoretical range of the indices lies between −17.5 and 17.5.

#### Learners’ Functional State

The degree to which students experienced *flow* (Csikszentmihalyi, 2000) and reacted with *worrying* was measured using the Flow Short Scale (FSS; Flow-Kurzskala) by Rheinberg et al. (2003). The FSS encompasses 10 items measuring the characteristics of flow, which allow for the computation of an overall score of flow (Cronbachs Alpha between α = 0.81 and α = 0.96) but also of two sub-scores (absorption by activity and fluency of performance). Furthermore, the FSS encompasses three additional items for measuring the degree to which one reacts with worrying in situations that are likely to evoke flow (Cronbachs Alpha between α = 0.80 and α = 0.90). The indicators of flow and worrying are generated by calculating the mean of a persons’ values on the 10 items measuring flow and the three items measuring worrying, respectively. The theoretical range of both indicators lies between 1 and 7, since the FSS uses a 7-point Likert scale with high values indicating high flow and worrying, respectively.

#### Interest in Continuing With the Learning Activity

Three items were constructed to measure the students’ prolonged interest in the learning activities (“I would like to learn more musical pieces of body percussion,” “I would be happy to have another lesson like this,” “I am happy not to have to learn more rhythms anymore”). A 7-point Likert scale ranging from “fits not at all” to “fits exactly” was used. The scale showed good internal consistency (Cronbachs Alpha α = 0.85). The indicator of interest in the learning activity is generated by calculating the mean of a persons’ values on the three items. Its theoretical range lies between 1 and 7 with high values indicating high interest.

## Results

### Changes in Relative Autonomous Motivation

**FIGURE 1:**
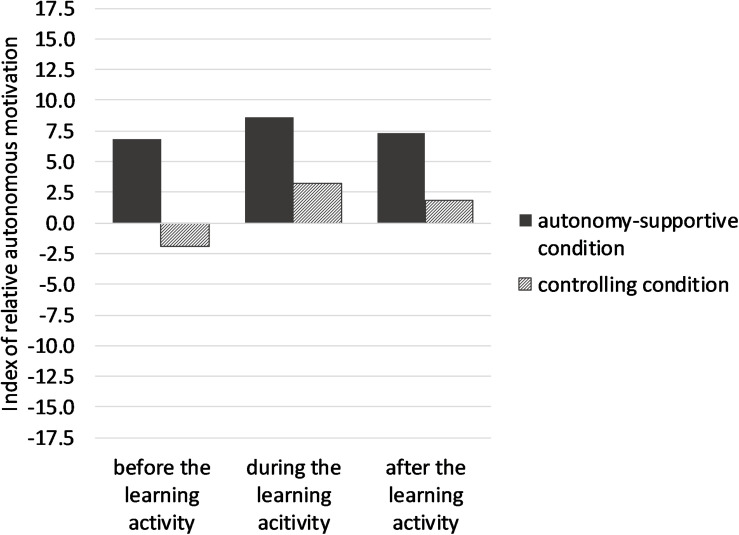
Averaged indices of relative autonomous motivation of students before, during and after the learning activity in the autonomy-supportive and the controlling condition.

We tested whether relative autonomous motivation increased in the course of the execution of the learning activity and whether this increase was moderated by instructional style. For this purpose, the index of relative autonomous motivation was subjected to a 3 (time of measurement: before, during, and after the learning activity) × 2 (instructional style: autonomy-supportive vs. controlling) mixed ANOVA (see Figure 1). Time of measurement constitutes the within-subject variable, instructional style constitutes the between-subject variable. For this analysis and all following statistical tests in this study we employed the conventional significance level of *p* < 0.05.

Assumptions of sphericity were violated for time of measurement as indicated by Mauchly’s test, *X*^2^(2) = 8.22, *p* = 0.016, *ε* = 0.953. Therefore, the degrees of freedom were corrected using Greenhouse–Geisser estimates of sphericity. There was a significant effect of time of measurement on relative autonomous motivation, *F*(1.906,312.626) = 11.15, *p* < 0.001, $\eta_{\text{p}}^{2}$ = 0.064. Contrast analyses showed that autonomous motivation was relatively higher during the learning activity than before the learning activity, *F*(1,164) = 23.85, *p* < 0.001, *d* = 0.76 and higher after the learning activity than before the learning activity, *F*(1,164) = 6.74, *p* = 0.01, *d* = 0.40. Thus, relative autonomous motivation increased in the course of the execution of the learning activity.

The main effect of instructional style on relative autonomous motivation was significant, too, *F*(1,164) = 31.05, *p* < 0.001, $\eta_{\text{p}}^{2}$ = 0.16. In the autonomy-supportive condition, relative autonomous motivation averaged over the times of measurement was higher (*M* = 7.57, *SD* = 8.00) than in the controlling condition (*M* = 1.14, *SD* = 9.98). Thus, an autonomy-supportive instructional style enhanced the relative autonomous motivation of students when being compared to the effects of a controlling instructional style.

The interaction effect between time of measurement and instructional style was significant, too, *F*(1.906,312.626) = 3.293, *p* = 0.041, $\eta_{\text{p}}^{2}$ = 0.02. As can be seen from Figure 1 the interaction between time of measurement and experimental condition can be classified as an ordinal interaction according to Leigh and Kinnear (1980). In order to analyze this finding in more detail, the differences between levels of a factor were examined separately for each level of the other factor by means of *t*-tests with Bonferroni–Holm-correction. First, changes in relative autonomous motivation in the course of the learning activity were considered separately for the two experimental conditions. Secondly, differences in relative autonomous motivation between students instructed in an autonomy-supportive style versus in a controlling style were analyzed separately for the three times of measurement.

In the controlling condition, relative autonomous motivation was higher during the learning activity (t2: *M* = 3.15, *SE* = 1.03) than before the learning activity (t1: *M* = −1.61, *SE* = 0.77), *t*(100) = 5.357, *p* < 0.001, *d* = 1.07. Relative autonomous motivation was also higher after the learning activity (t3: *M* = 1.95, *SE* = 1.11) than in t1, *t*(98) = 3.632, *p* = 0.001, *d* = 0.74 Thus, against expectations an increase in relative autonomous motivation occurred in the controlling condition. In the autonomy-supportive condition, relative autonomous motivation only descriptively was higher in t2 (*M* = 8.67, *SE* = 0.96) than in t1 (*M* = 6.73, *SE* = 0.74), but this difference was not significant, *t*(69) = 1.808, *p* = 0.075 (one-tailed), *d* = 0.44. The difference in relative autonomous motivation between t1 and t3 (*M* = 7.32, *SE* = 1.15) was not significant, too, *t*(67) = 0.396, *p* = 0.347 (one-tailed), *d* = 0.10. Thus, in contrast to Hypothesis 1, relative autonomous motivation did increase significantly in the course of the learning activity in the controlling condition, but did not increase significantly in the autonomy-supportive condition.

For analyzing the effect of instructional style on relative autonomous motivation in more detail, three independent *t*-tests were conducted, one for each time of measurement. In all three situations, students were more autonomously motivated when being instructed in an autonomy-supportive style than when being instructed in a controlling style: t1: *t*(170) = 7.54, *p* < 0.001, *d* = 1.16; t2: *t*(166.68) = 3.917, *p* < 0.001, *d* = 0.58; t3: *t*(165) = 3.27, *p* = 0.001, *d* = 0.51.

In line with Hypothesis 2, students instructed in an autonomy-supportive style were more autonomously motivated at all three times of measurement than students instructed in a controlling style.

### Relative Autonomous Motivation, Instructional Style and Students’ Functional State

In order to examine the relationship between students’ relative autonomous motivation and their functional state during the execution of the learning activity, the correlation between relative autonomous motivation during the learning activity and the flow experience of students as well as their degree of worrying was computed. As expected (Hypothesis 3), there was a significant positive correlation between relative autonomous motivation and experiencing flow, *r* = 0.42, *p* < 0.001. Not in line with expectations, relative autonomous motivation during the learning activity also correlated positively with worrying, *r* = 0.21, *p* = 0.007.

In order to examine the effect of an autonomy-supportive instructional style compared to a controlling instructional style on students’ functional state during the learning activity, two *t*-tests for independent samples with Bonferroni–Holm-correction were executed with flow and worrying as the dependent variables, respectively. As expected (Hypothesis 4), students in the autonomy-supportive condition experienced flow to a higher degree (*M* = 4.98, *SE* = 0.11) than students in the controlling condition (*M* = 4.43, *SE* = 0.11), *t*(170) = 3.31, *p* = 0.002, *d* = 0.51. However, the difference in worrying between students in the autonomy-supportive condition (*M* = 2.94, *SE* = 0.19) and students in the controlling condition (*M* = 3.11, *SE* = 0.17) was not significant, *t*(170) = 0.67, *p* = 0.502, *d* = 0.10.

### Effects of Relative Autonomous Motivation and Instructional Style on Students’ Interest in Continuing With the Learning Activity

A multiple linear regression was performed in order to test the hypothesis that interest in continuing with the learning activity is predicted by the relative autonomous motivation of students during the learning activity and instructional style. The model with relative autonomous motivation and instructional style as predictors for interest was significant, *F*(2,168) = 49.95, *p* < 0.001, with *R*^2^ = 0.37. As can be seen in Table 1, relative autonomous motivation and experimental condition were both significant predictors of interest, which is in line with Hypothesis 5.

**TABLE 1:** Linear model of instructional style and relative autonomous motivation as predictors of interest.

	*b*	*SE B*	β	*p*
Constant	4.27 (3.95, 4.57)	0.15		*p* = 0.001
Instructional style	0.52 (0.07, 1.05)	0.23	0.14	*p* = 0.026
Relative autonomous motivation during learning activity	0.10 (0.08, 0.12)	0.01	0.56	*p* = 0.001

*In parentheses 95% bias corrected and accelerated confidence intervals are reported. Confidence intervals and standard errors are based on 1,000 bootstrap samples.*

## Discussion

### Summary and Evaluation of the Hypotheses

In this study, short-term motivational dynamics in other-initiated learning situations were investigated. Results showed that relative autonomous motivation increased in the course of the execution of an other-initiated learning activity. Referring to the action-based model of cognitive dissonance theory (Harmon-Jones et al., 2015) such an increase in relative autonomous motivation was expected. According to this model, people are motivated to resolve states of dissonance in order to be able to execute actions efficiently. Executing learning activities due to the request of another person potentially induces dissonance because of the potential to frustrate the need for autonomy. Finding reasons in such a situation and/or stressing their importance for executing the learning activity that are experienced as stemming from one’s self (Ryan, 1993) and/or neglecting reasons and/or stressing their importance less that are experienced as not stemming from one’s self – thus increasing one’s relative autonomous motivation – may reduce feelings of conflict and support an efficient execution of actions. Thus, the finding of an increase in relative autonomous motivation is in line with predictions of the action-based model of cognitive dissonance.

However, based on self-determination theory and in line with assumptions of cognitive dissonance theory as well (Linder et al., 1967) it was expected that this increase in relative autonomous motivation would more likely occur in situations in which heteronomy is not dominant. Thus, an increase in relative autonomous motivation was expected only in autonomy-supportive but not in controlling situations. Yet, against our hypothesis, the increase in relative autonomous motivation was only significant in the controlling condition. Potential explanations for this unexpected finding will be outlined in the following.

First, it must be kept in mind that the absolute level of relative autonomous motivation was higher in students instructed in an autonomy-supportive style than in students instructed in a controlling style at all times of measurement, especially before the execution of the learning activity. An increase in relative autonomous motivation simply might be more pronounced when starting from a lower level of relative autonomous motivation than when starting from a higher level.

Second, the unexpected finding of an increase of relative autonomous motivation in a controlling situation might be explained by features of the learning task. Students were novices with regard to body percussion. Initially, learning body percussion might offer novices great possibilities to feel growth of competence. Perceiving oneself as competent increases the likelihood for intrinsic motivation to flourish and internalization to take place (Vallerand, 1997; Ryan and Deci, 2000b). The need for competence might have been satisfied to such a strong extent by means of this learning activity that the importance of the instructional style (viz., the need for an autonomy-supportive instructional style) for internalization and intrinsic motivation to be fostered might have been reduced. If growth of competence is smaller during a learning activity, the degree of autonomy-support might be crucial for whether an increase in relative autonomous motivation will take place or not. Hence, in future studies it should be investigated whether the effect of the execution of a task on changes in the quality of motivation and its interaction with instructional style depends on features of the learning task.

Third, the ordinal interaction between condition (autonomy-supportive vs. controlling) and time of measurement might be explained by the possibility that the assumptions we had about (a) how students would experience other-initiated learning activities and (b) how important voluntariness would be for processes of dissonance reduction apply to a smaller degree to the kind of learning activity we employed in the present study than expected. Our hypothesis that dissonance would only be reduced in autonomy-supportive conditions rested on two assumptions. Assumption 1 was that a situation in which a learning activity is initiated by another person would at least initially be to some extend experienced as other-determined, regardless of the instructional style used. The reasoning was that if an individual is asked to execute a learning behavior requested by another person, the most salient reason was expected to be that the impetus to execute the activity was given by the other person. Therefore, both experimental conditions were expected to be experienced to some degree as other-determined situations and consequently as situations in which dissonance was expected to emerge. However, given that students’ index of relative autonomous motivation differed significantly between the two experimental conditions in t1 and was positive in the autonomy-supportive condition and negative in the controlling condition, the validity of Assumption 1 is questionable. The data instead suggest that initially only the controlling condition was experienced as other-determined whereas the autonomy-supportive condition was not. Thus, dissonance might have only been evoked in the controlling but not in the autonomy-supportive condition. Accordingly, processes of dissonance reduction would mainly be expected in the controlling condition.

The second assumption underlying our initial hypothesizing was that in other-determined situations, dissonance would only be reduced if some experience of voluntariness was given (cf. Linder et al., 1967). This assumption can also be put into question. Although the controlling instructional style supposedly hampered feelings of voluntariness, relative autonomous motivation nevertheless increased during the execution of the learning activity in this experimental condition. Thus, voluntariness does not seem to be a necessary condition for dissonance to be reduced for the kind of activity employed in this study. The critical difference might be that studies in which voluntariness proved to be of importance for dissonance reduction focused on counter-attitudinal behaviors (e.g., Cohen et al., 1959; Vaidis and Gosling, 2011) whereas the learning task in the present study is not considered to be counter-attitudinal *per se*.

According to the action-based model commitment for an action leads people to be motivated to execute the behavior effectively (Harmon-Jones et al., 2015). Therefore, the attitude toward the activity individuals have committed to – be it based on controlling or based on autonomy-supportive instructions – will be changed to support the effective execution of that behavior, but only if change due to dissonance is needed. Hence, if Assumptions 1 and 2 underlying our initial hypothesis of an interaction between instructional style and time of measurement do not apply to the learning activity employed in the present study, this might explain why relative autonomous motivation increased in the controlling condition. Students in the controlling condition changed the dissonance-arousing situation of being in an other-determined, controlling situation to a less controlling situation, in which the requested activity is carried out due to increasingly autonomous and less controlled motivation. In contrast, in the autonomy-supportive condition, right from the outset high levels of relative autonomous motivation were triggered. Therefore, dissonance might not have been elicited in the autonomy-supportive condition and, accordingly, processes of dissonance reduction are unlikely. Instead, since relative autonomous motivation was higher at all times of measurement in the autonomy-supportive condition compared to the controlling condition it might be reasoned that actual internalization took place in the autonomy-supportive condition to a greater extent than in the controlling condition.

Summarized, these interpretations of the results suggest that the increase of relative autonomous motivation in the controlling condition could be mainly traced back to the reduction of dissonance while the higher levels of relative autonomous motivation in the autonomy-supportive condition compared to the level in the controlling condition could be primarily traced back to the initiation of internalization and flourishing of intrinsic motivation.

Results concerning students’ functional state differed depending on whether the experience of flow or worrying were used as indicators of students’ functioning. Results with regard to the experience of flow – an indicator of a positive functional state – were in line with Hypothesis 3 and 4. Students’ relative autonomous motivation correlated positively with experiencing flow and students in the autonomy-supportive condition experienced flow significantly stronger than students in the controlling condition. The correlation between relative autonomous motivation and flow can be classified as a medium-sized effect (Cohen, 1988) and the mean value of flow indicates that students confirmed the experience of flow (*M* > 4 on a 7-point Likert scale). Results with regard to worrying – an indicator of a negative functional state – were not as expected. Relative autonomous motivation also correlated positively (though weakly) and not negatively with students’ degree of worrying and there were no significant differences in worrying between the two experimental conditions, so students in the autonomy-supportive condition did not worry less than students in the controlling condition. It must be kept in mind though that in both experimental conditions the mean value of worrying of students still indicated rejection of worrying (*M* < 4 on a 7-point Likert scale). Concerning the interpretation of the unexpected positive correlation between relative autonomous motivation and worrying, in our view, it is questionable, whether it actually points to an unfavorable relationship between relative autonomous motivation and the functional state of students. This is, because (a) the mean values of worrying indicated that students did not worry, (b) there were no significant differences in worrying between the two experimental conditions although students were clearly more autonomously motivated in the autonomy-supportive condition, and (c) the results with regard to flow are consistent with Hypothesis 3. Because of the correlational nature of this data, no conclusion can be made about the causal relationship between relative autonomous motivation and the functional state of students. Clearly though, it can be reasoned that giving instructions in an autonomy-supportive style compared to a controlling style is facilitative of the functional state of learners since these conditions were experimentally manipulated in this study. In future studies it would be worth investigating the nature of the relationship of relative autonomous motivation and flow and whether for example the effect of instructional style on the functional state of students is mediated by its effect on relative autonomous motivation.

In line with our hypotheses, students’ interest in continuing with the learning activity later on was predicted by relative autonomous motivation during the learning activity and instructional style. This result is further experimental evidence for the beneficial effects that an autonomy-supportive instructional style has on students’ prolonged interest for learning activities compared to a controlling style. It also highlights the importance of the quality of motivation during the execution of a learning activity for the likelihood that the learning activity will be carried out again in the future.

### Processes Underlying Motivational Change

In this study, two processes for motivational changes taking place during the execution of an other-initiated learning activity were proposed. Based on the action-based model of cognitive dissonance, reduction of dissonance was considered to explain changes in relative autonomous motivation. Based on self-determination theory, internalization was considered to explain changes in relative autonomous motivation. Our study was not designed to differentiate these interpretations on the empirical level. *Post hoc*, however, the results of our study suggest that in autonomy-supportive situations an increase in relative autonomous motivation during an other-initiated learning activity would be based on internalization rather than on reduction of dissonance while in controlling situations the increase is more likely based on reduction of dissonance. Further theoretical approaches to explain increases in relative autonomous motivation could also be applicable, such as the activation of an interest-creating discovery module due to dynamic self-regulation (Iran-Nejad and Chissom, 1992). Future studies should test on the empirical level how these approaches contribute to explaining motivational changes.

The results of the present study stand in contrast to findings indicating a decrease in students’ relative autonomous motivation over longer periods of time and with regard to certain school subjects or school education in general. We conclude that long- and short-term motivational changes are regulated by different processes. In the hierarchical model of intrinsic and extrinsic motivation (Vallerand, 1997), motivation is conceptualized at three different levels of specificity, the global, the contextual and the situational level. In our study, motivation is investigated at the situational level in a fine-grained way by measuring motivation three times in situations related to the same activity. Studies, in which motivational changes are examined in long periods of time, focus on the contextual level of the hierarchical model (Vallerand, 1997). It is also assumed in this model that there are different determinants for motivation on the different levels of analysis (Vallerand, 1997). Situational motivation is thought to result from the evaluative perception of situational factors by the individual whereas contextual motivation is thought to result from the evaluative perception of contextual factors by the individual. Therefore, from this theoretical viewpoint, differences in the absolute level in situational and contextual motivation are possible, even so it is assumed that motivation on higher levels can affect the motivation on lower levels and vice versa. Likewise, it is reasonable to suspect that changes in relative autonomous motivation on the situational and on the contextual level can be traced back to different processes and that the direction of change might differ.

On the contextual level, motivation relates to school as an institution or school subjects. Motivation here potentially pertains to a broad array of behaviors and students probably indicate their motivation rather in retrospect and detached from the experience of executing a particular activity. On the situational level, as measured in our study, students indicate their motivation in the moment and based on the experience of being in a specific situation and executing a particular activity. Therefore, the statements made by the individual about their contextual motivation are based on reflections on different information than statements made about their situational motivation. Especially, the immediacy of the need to act and thus the immediacy of the need to reduce dissonance differs on the two levels. On the situational level, the individual actually executes an activity and, therefore, is motivated to execute the behavior effectively which requires a reduction of dissonance. On the contextual level such an immediate need to reduce dissonance by increasing relative autonomous motivation is not given, since there is no immediate demand for action. Accordingly, rather than reacting with increasing relative autonomous motivation in response to controlling contexts, the individual might reflect on the experience of the given context and the individuals’ motivation changes accordingly without any compensatory dissonance reduction. Consequently, the decrease in relative autonomous motivation measured at the contextual level has for example been ascribed to students perceiving their teachers as becoming less autonomy-supportive in the course of their school years (e.g., Gillet et al., 2012; Martinek et al., 2016). On the situational level in contrast, it seems that the frustration of the need for autonomy and the resulting experience of dissonance can be compensated to some extent by processes of dissonance reduction.

### Practical Implications

The results provide further evidence on desirable effects of an autonomy-supportive instructional style as it (a) increased relative autonomous motivation, (b) fostered a positive functional state in learners and (c) increased students’ interest in continuing with an executed learning activity. Since relative autonomous motivation increased in the course of the execution of an other-initiated learning activity, the results also point to beneficial effects of executing a learning activity for fostering autonomous motivation. Thus, it seems to be important to prompt students to actually execute a planned learning activity, instead of only showing or explaining this activity to them. Especially in the controlling situation, relative autonomous motivation increased after the threshold from intention to action was transcended and learners got involved in the learning activity. Whether such changes in motivation are long-lasting or whether for long-lasting changes autonomy-support is needed, is unknown and should be focused in future experiments.

The results also point to the significance that the quality of motivation as differentiated in self-determination theory has for the functional state of learners and their prolonged interest for learning activities. Therefore, and also because these results fit in with related findings in the field (e.g., Evans and Bonneville-Roussy, 2016), it appears to be a valuable goal to inform practitioners about the importance of the quality of their students’ motivation in terms of its degree of autonomy and support their understanding of how to foster beneficial types of motivation in educational settings. This is even more important, since autonomy-support seems to have positive effects on more far reaching aspects of students’ life than discussed in this article like their kind of passion for the field of activity (Bonneville-Roussy et al., 2013) or their well-being (Bonneville-Roussy et al., 2020).

The results of this study might also be of interest for practical applications in research. The study showed that motivation changes in the course of the execution of an activity. If the assessment of effects of motivation in an experimental setting are the matter of interest it might be of importance at what point in time motivation is measured.

Finally, the study also was informative with regard to the measurement of motivation. Buff (2001) and Buff et al. (2010) introduced an approach to measure motivation by asking participants to write down reasons in their own words for engaging themselves in tasks. This approach complies very well with the claim by Vallerand (1997) that assessing motivation requires measuring motivation by recording the concrete reasons for engaging in a certain activity, whereas oftentimes actually the determinants or consequences of motivation are taken as measures of motivation. The recorded reasons are assigned to categories. The categories are either assigned to the overarching types of intrinsic, self-determined and other-determined motivation (Buff, 2001) or values are ascribed to each category expressing to what extent reasons falling into a particular category signify autonomous regulation (Buff et al., 2010). By this, an individuals’ motivational orientation (Buff, 2001) or an individuals’ degree of autonomous regulation can be determined (Buff et al., 2010). In the present study, a slightly extended way of processing recorded reasons was used. Participants were not only asked to name reasons but also to rate the importance of each of their reasons. This data was combined in the index of relative autonomous motivation so that qualitative information (what kind of reason) as well as quantitative information (how important) contribute to this index. This procedure proofed to be sensitive to detect changes in relative autonomous motivation taking place in a short period of time. It therefore seems to be an applicable alternative to widely used standardized measures like the Academic Motivation Scale (Vallerand et al., 1993) or the Academic Self-Regulation Questionnaire (Ryan and Connell, 1989) in order to measure the level of relative autonomous motivation, especially if precise knowledge about which reasons are of importance in that domain or for a specific activity is lacking.

### Limitations

Some limitations of this study need to be considered. Only one learning activity was used: body percussion and vocussion. Thus, the generalizability of the results is limited. What characterizes the learning activity employed here is that students make music actively in a group, no special musical training is required to be able to participate in this activity and there are opportunities for the individual to increase the level of difficulty according to the individuals’ level of ability. On a more general level, the learning activity implemented in this study was characterized by the following features: it was initiated by another person, a reasonable learning progress could be expected within one lesson, the learning progress is partially based on the learner’s own activity, and the activity inherently provides feedback for the learners. Although we implemented this type of learning in a music lesson, activities with such features can, in principle, be created also in other school subjects. Therefore, we would expect that the results of this study should be generalizable to comparable learning activities in subjects other than music, too. Future research should test this assumption, though, and extend knowledge about motivational changes by using different types of learning activities and also by varying the domain of learning. In this way, light could be shed on the question whether and if so which characteristics of the learning task influence motivational changes and the processes underlying these changes.

The experimental situation was only manipulated with regard to its degree of autonomy-support. Other situational factors that potentially influence changes in relative autonomous motivation were not manipulated or controlled for. Such other factors might for example be the induced mind-set (Gollwitzer and Bayer, 1999) or degree of motivational conflict (Grund, 2013) triggered by the situation. An actional mind-set or a low degree of motivational conflict could support learners to focus on the task at hand, which in turn might influence relative autonomous motivation. Including additional situational factors in future studies could help to determine further conditions and processes underlying motivational dynamics.

The sample of this study consisted of young secondary school students. In order to test whether the results of this study can be generalized across different age groups future studies should include participants of different age groups.

## Data Availability Statement

The datasets generated for this study are available on request to the corresponding author.

## Ethics Statement

Based on the declaration in the ethical guidelines of the Federation of the German Psychologists’ Associations (2016) as well as the American Psychological Association (2017) that psychologists may dispense with informed consent if participation is reasonably not harmful to subjects, research refers to common teaching situations in educational settings, and involves only anonymous questionnaires, written informed consent from the participants’ legal guardian/next of kin was not required to participate in this study. This application of the ethical guideline to the study was independently confirmed by the participating schools who were informed about the procedures and goals of the study and approved it. Participants were informed about the procedure of the study beforehand and were thoroughly debriefed about the experimental manipulation after the study. Ethical review and approval was not required for the study on human participants in accordance with the local legislation and institutional requirements.

## Author Contributions

PH and KH designed the study. KH collected the data. PH analyzed the data. PH and SD wrote the manuscript. All authors read and approved the submitted version.

## Conflict of Interest

The authors declare that the research was conducted in the absence of any commercial or financial relationships that could be construed as a potential conflict of interest.

## Appendix: Categorization of Reasons Named by Students

**TABLE A1:** Categories of reasons for engaging in a learning activity with explanations and the reference value of each category (scaled from −2.5 to 2.5).

Category	Explanation: the learning activity is executed because…	Reference value
(1) Positive emotions	… positive emotions are associated with the activity.	2.5
(2) Interest	… interest in the activity is explicitly stated.	2.5
(3) Gain in competence	… the learner aims to understand, to master, or, generally, to learn something.	1.9
(4) Testing one’s abilities	… the learner wants to find out whether he or she is able to master the task at hand.	1.1
(5) Usefulness	… general or personal usefulness is ascribed to the topic dealt with.	0.9
(6) Positive self-diagnosis	… the learner ascribes high competencies with regard to the activity to him- or herself.	0.9
(7) Negative self-diagnosis	… the learner ascribes limited competencies with regard to the activity to him- or herself.	0.7
(8) Desire for improvement	… the learner wants to improve him- or herself, his or her grades, or the like.	0.7
(9) Relevance	… general or personal relevance is ascribed to the topic dealt with.	0.5
(10) Being “good”	… the learner desires to be “good” or to have good grades, or because being “good” or having good grades are means for the learner to receive positive consequences.	−0.1
(11) Helping others	… the learner wants to help others with his or her actions.	−0.3
(12) Finishing activities	… the learner wants to finish the activity he or she had begun with.	−0.5
(13) Not being “bad”	… the learner desires not to be “bad” or to have bad grades, or because not being “bad” or not having bad grades are means for the learner to prevent negative consequences.	−0.9
(14) Gratifications, sanctions, pressure, coercion, and expectations	… the learner aims to receive or prevent tangible or social gratifications or sanctions, respectively, or wants to meet expectations of others or acts due to pressure or coercion.	−2.5
(15) Rest	… of reasons not covered by the 14 categories above.	
